# Application of the direct in-scope suction technique in antegrade flexible ureteroscopic lithotripsy for the removal of a large ureteric calculus in a kidney transplant recipient: A case report

**DOI:** 10.1016/j.eucr.2024.102663

**Published:** 2024-01-21

**Authors:** Sucha Kritsing, Kraipith Udomsombatmeechai, Ornnicha Prohsoontorn, Nopparuj Sangnoppatham, Chinnakhet Ketsuwan

**Affiliations:** a Division of Urology, Department of Surgery, Faculty of Medicine Ramathibodi Hospital, Mahidol University, Bangkok, Thailand; b Excellent Center of Organ Transplantation, Ramathibodi Hospital, Mahidol University, Bangkok, Thailand

**Keywords:** Antegrade flexible ureteroscopy, DISS, Kidney transplant, Ureteric calculus

## Abstract

The occurrence of a large ureteric calculus in a transplanted kidney, originating from a donor, is a rare but significant complication. It poses risks such as urinary obstruction, septicemia, and potential loss of allograft function. In this case, we report our first use of the direct in-scope suction technique during antegrade flexible ureteroscopy lithotripsy. This method successfully removed a donor-derived ureteric calculus in a kidney transplant recipient. The procedure resulted in complete stone removal, and the patient experienced a favorable postoperative recovery without additional adverse events.

## 1 Introduction

Kidney transplantation (KT) is a pivotal milestone in the evolution of renal replacement therapies and is the optimal therapeutic choice for individuals with end-stage kidney disease. Within the spectrum of post-transplant complications, kidney allograft urolithiasis emerges as a critical concern, including urinary obstruction, infection, and the potential for graft loss.^1^ The severity of these concerns is heightened when calculi obstruct the transplant ureter. Urolithiasis following KT is relatively uncommon; the incidence of calculi in renal transplant recipients fluctuates between 0.2 % and 5.7 %, typically remaining below the 1 % threshold.^2^ The therapeutic strategies for addressing lithiasis in kidney transplant recipients closely mirror those employed for individuals with a solitary kidney. These strategies encompass active monitoring, extracorporeal shock wave lithotripsy, flexible ureteroscopic lithotripsy (FURSL), and percutaneous nephrolithotomy.^3^, ^4^, ^5^

The growing use of high-power lasers during FURSL has prompted endourologists to investigate methods for aspirating the fine dust produced, which is frequently associated with postoperative complications, especially in immunocompromised hosts. The direct in-scope suction (DISS) technique has emerged as a novel approach to advocate for concurrent or sequential suction during or following laser lithotripsy. In this paper, we present what we believe to be a pioneering clinical case in which the DISS technique was integrated with antegrade FURSL to successfully remove a sizeable ureteric calculus in a kidney transplant recipient.

## 2 Case report

A 50-year-old female patient with an idiopathic etiology of end-stage kidney disease underwent continuous ambulatory peritoneal dialysis, receiving five cycles daily for five years. A pretransplant urologic evaluation indicated no abnormalities, and she was scheduled for a transplant from a primary deceased donor. The donor, a 63-year-old male, had a medical history of right intracranial hemorrhage but no known pre-existing urologic conditions. Unfortunately, a comprehensive assessment for donor-gifted kidney transplant lithiasis, including routine ultrasonography and urinalysis, could not be conducted before organ procurement due to equipment limitations at the rural hospital. During KT, the kidney graft was positioned in the right iliac fossa. Surgical details included an end-to-side anastomosis of the renal vein to the external iliac vein and the renal artery to the external iliac artery using 6/0 Prolene running sutures. A 4.8Fr double-J stent was inserted into the ureter, employing the Lich-Gregoire technique for ureterovesical anastomosis with interrupted 5/0 polyglyconate sutures. The patient's immediate postoperative period was unremarkable, leading to her discharge with a creatinine level of 1.78 mg/dL.

**Fig. 1:**
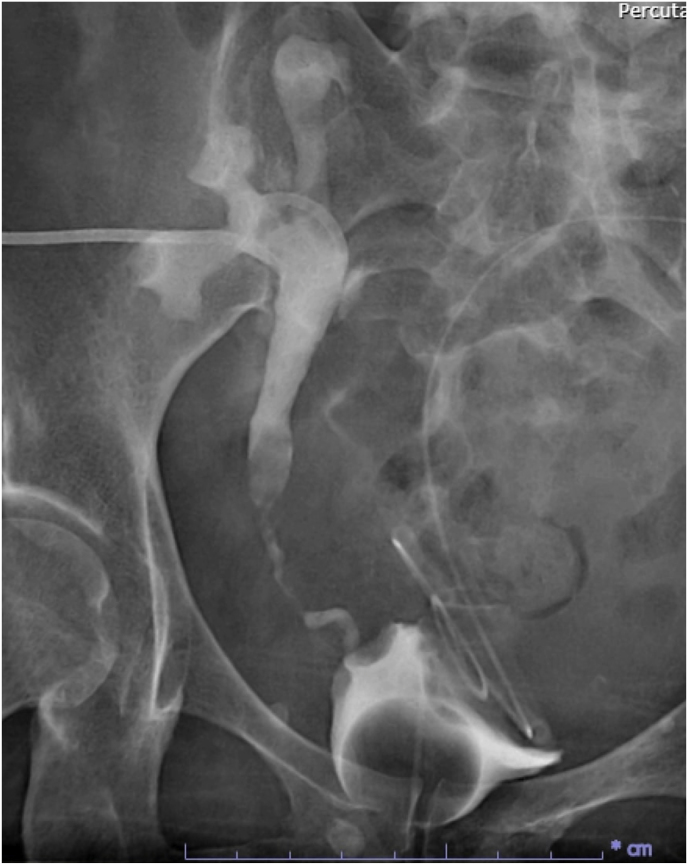
Antegrade pyelography revealing a mid-ureteric calculus in a kidney allograft.

The double-J stent was cystoscopically removed two weeks post-transplant. However, she presented the following day to the emergency department with anuria and abdominal discomfort. Laboratory investigations revealed a creatinine increase to 4.61 mg/dL. Renal ultrasound showed mild hydronephrosis in the transplanted kidney, although the parenchyma remained intact. Given the urgency of the situation, an interventionist was consulted to perform a percutaneous nephrostomy for immediate alleviation of the urinary obstruction. Subsequent antegrade pyelography revealed a 2 cm filling defect in the mid-ureter, suggestive of a ureteric calculus (Fig. 1). After a thorough discussion of the surgical options with the patient, a decision was made to proceed with antegrade FURSL and use the DISS technique for effective stone removal.

**Fig. 2:**
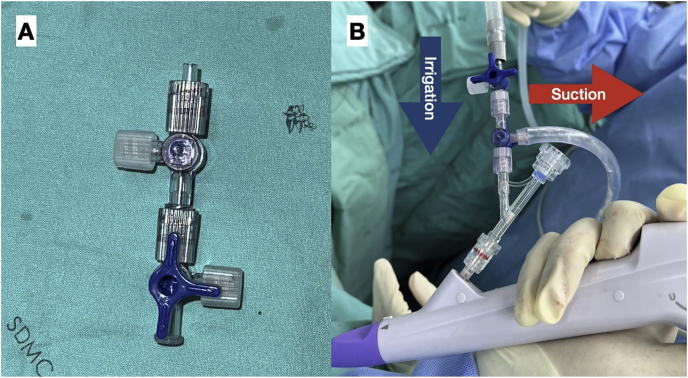
A, Assembly adapter for the DISS device; B, Operating the scope with the attached adapter.

**Fig. 3:**
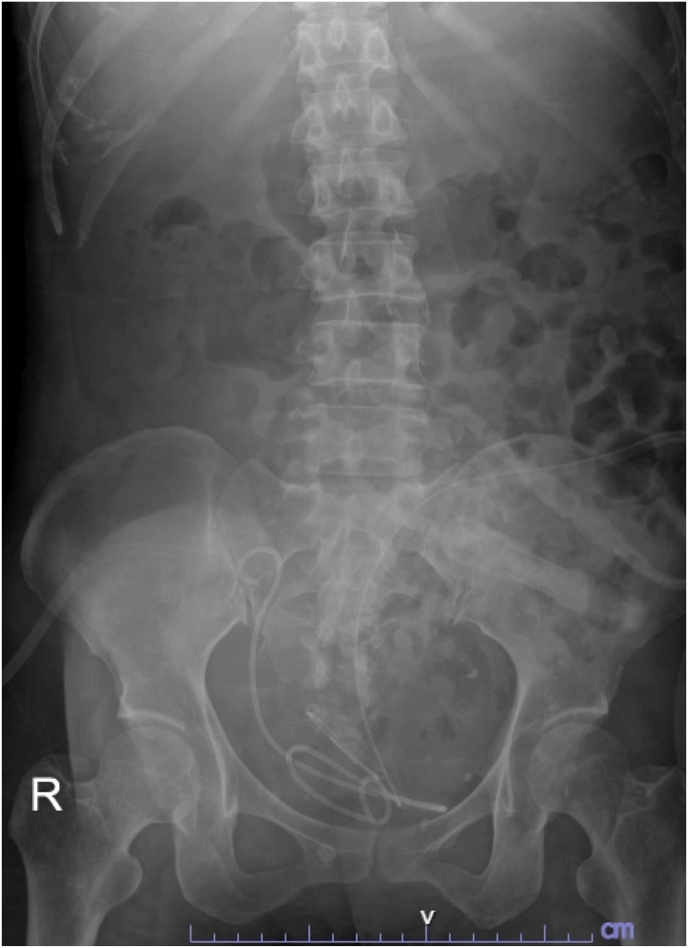
Postoperative abdominal radiography.

To facilitate the efficacy of the DISS technique, we connected two 3-way connectors, which allowed one inlet to be dedicated for suction tubing and the other for irrigation (Fig. 2A). This versatile attachment was then seamlessly integrated into the working inlet of any flexible ureteroscope (Fig. 2B). During the initial phase of administering general anesthesia, prophylactic intravenous cefuroxime was administered, and the patient was strategically positioned in the lithotomy posture. Access to the lower pole of the right iliac fossa was achieved percutaneously, tracing the path of the existing percutaneous nephrostomy tube. Antegrade pyelography provided imaging of the pyelocalyceal system and identified the ureteric stone's location. Subsequently, a hybrid guidewire was introduced into the renal pelvis via a nephrostomy pigtail catheter. Following guidewire placement, the nephrostomy pigtail catheter was removed, and a secondary guidewire was inserted using a 10F dual-lumen catheter. The tract was then dilated over the guidewire, facilitating the placement of a 14/16 Fr Clear Petra disposable nephrostomic sheath (Well Lead Medical Co.) under fluoroscopic guidance. The UROMAT E.A.S.I. pressure-controlled double-roller pump (Karl Storz, Tuttlingen, Germany) was utilized, maintaining a low pressure at 40 mmHg. The procedure continued with the introduction of an 8.4 Fr flexible ureteroscope (fURS) (Scivita Medical, Suzhou, China) through the access sheath. A pure dusting technique was employed using a 120 W Ho:YAG laser (Lumenis, San Jose, CA) with a 365 μm core laser fiber, set at 0.5 J and 30 Hz. The 3-way suction adapter was opened sequentially, either for aspirating fragments during a ‘snow-globe effect’ or intermittently for removing dust after lithotripsy. By maintaining the scope's alignment with the ureteric calculus, the generated dust was efficiently aspirated, allowing for the continuation of further lithotripsy procedures. After the procedure, a 6 Fr double-J stent and a nephrostomy tube were placed. The postoperative assessment included obtaining plain abdominal films. This was done to evaluate for residual stones and to ensure correct positioning of both the nephrostomy tube and the double-J stent (Fig. 3). The patient experienced a smooth postoperative recovery with a total surgical duration of 90 minutes and an estimated blood loss of 50 mL. The patient's creatinine level decreased to her baseline value of 1.5 mg/dL. The percutaneous nephrostomy tube was removed 5 days postoperatively, and the double-J stent was cystoscopically removed 3 weeks post-discharge. After removing the double-J stent at one month, we plan to perform a computed tomography (CT) scan to assess the presence of any residual ureteric stones and the resolution status of hydronephrosis. Due to financial limitations, analysis of the stone composition was not performed.

## 3 Discussion

Over the past decade, there has been a significant increase in global demand for organ transplantation. This surge is primarily driven by a rising incidence of vital organ failure, improved success rates, and significant advancements in post-transplant outcomes. However, this increasing demand stands in stark contrast to the inadequate supply of available organs, leading to a profound organ shortage crisis. To address this issue, some transplant centers have started to modify traditional donation criteria.^6^ Donors with incidentally identified asymptomatic renal and ureteral stones, commonly referred to as “donor-gifted lithiasis,” are increasingly being considered for organ donation, which is contingent upon the absence of recurrent stone formation and active biochemical disorders. This trend is supported by the demonstrated safety and manageability of graft lithiasis as well as the relatively low risk of lithiasis recurrence in such scenarios.^7^

To date, surgical treatment options for ureteric calculus in allografts predominantly involve minimally invasive approaches. Extracorporeal shock wave lithotripsy was previously a widely used method for treating small stones in kidney allografts. Nevertheless, its role has significantly diminished due to challenges in accurately localizing stones, as shock waves can be obstructed by an overlying bony pelvis. FURSL has seen significant improvements in terms of vision, durability, and maneuverability. It is a suitable option for allograft kidney stones with diameters of <1.5 cm. However, for larger stones with a diameter exceeding 2 cm or those of a complex nature, FURSL is not recommended as a first-line treatment modality.^8^ Percutaneous treatment of calculi located within the transplanted kidney is typically performed for stones >1.5 cm and has proven to be safe and effective. Moreover, additional refinements in technique, including minimally invasive percutaneous nephrolithotomy, which utilizes a smaller tract to decrease the risk of bleeding and tearing of the renal cortex, have also gained popularity.^9^, ^10^, ^11^ Our patient has a large ureteric calculus located in the mid-part of the transplant ureter, and the patient underwent postoperative ureteroneocystomy only 3 weeks ago. The retrograde approach poses a high risk of anastomotic damage. Therefore, antegrade ureteroscopy through a minimally invasive percutaneous tract is a better option.

The laser lithotripsy procedure aims to thoroughly fragment stones regardless of location, which minimizes complications and ensures the complete removal of fragments to prevent significant residual stones.^12^ When residual stones are left post-operation, the risk of hospital admission varies between 14 % and 19 %, with the risk of requiring further intervention ranging from 12 % to 35 %.^13^ This risk escalates in cases involving a solitary kidney or post-KT. In the diagnostic imaging of residual stones, available modalities include plain radiography, ultrasound, and CT scans. However, plain radiography and ultrasound show lower sensitivity (48–63 %), potentially leading to the missed detection of smaller residual stones. Conversely, CT scans offer superior sensitivity and specificity but involve increased radiation exposure.^14^ Integrating suction techniques, particularly the DISS technique, to improve outcomes has been advanced. This innovation adheres to the foundational principles of traditional FURSL, allowing endourologists to enhance their existing practice with a straightforward modification.^15^ The 3.6 Fr working channel of the scope serves as an effective suction conduit, enabling the efficient aspiration of fine dust particles without causing blockages. To our knowledge, this is the first case to report the application of the DISS technique in treating large ureteric calculi in allograft patients.

## 4 Conclusion

We successfully treated a large mid-ureteral calculus by applying the DISS technique in the treatment of large ureteric calculi in allograft patients. Integrating a suction system not only reduces the likelihood of postoperative complications but also improves visibility during the procedure, thereby speeding up the process**.**

## Declaration of competing interests

None.

## CRediT authorship contribution statement

**Sucha Kritsing:** Writing – original draft, Data curation, Conceptualization. **Kraipith Udomsombatmeechai:** Methodology, Investigation. **Ornnicha Prohsoontorn:** Project administration, Investigation. **Nopparuj Sangnoppatham:** Formal analysis, Data curation. **Chinnakhet Ketsuwan:** Writing – review & editing, Validation, Supervision.
